# Coping with the Challenges of Abiotic Stress in Plants: New Dimensions in the Field Application of Nanoparticles

**DOI:** 10.3390/plants10061221

**Published:** 2021-06-15

**Authors:** Vishnu D. Rajput, Tatiana Minkina, Arpna Kumari, Harish, Vipin Kumar Singh, Krishan K. Verma, Saglara Mandzhieva, Svetlana Sushkova, Sudhakar Srivastava, Chetan Keswani

**Affiliations:** 1 Academy of Biology and Biotechnology, Southern Federal University, 344090 Rostov-on-Don, Russia; tminkina@mail.ru (T.M.); msaglara@mail.ru (S.M.); terra_rossa@mail.ru (S.S.); 2 Department of Botanical and Environmental Sciences, Guru Nanak Dev University, Amritsar 143005, India; arpnabot.rsh@gndu.ac.in; 3 Plant Biotechnology Laboratory, Department of Botany, Mohan Lal Sukhadia University, Udaipur 313001, India; harish.botany1979@gmail.com; 4 Centre of Advanced Studies in Botany, Institute of Science, Banaras Hindu University, Varanasi 221005, India; vipinks85@gmail.com; 5 Key Laboratory of Sugarcane Biotechnology and Genetic Improvement, Guangxi Academy of Agricultural Sciences/Sugarcane Research Center, Chinese Academy of Agricultural Sciences, Nanning 530007, China; drvermakishan@gmail.com; 6 Plant Stress Biology Laboratory, Institute of Environment and Sustainable Development, Banaras, Banaras Hindu University, Varanasi 221005, India; sudhakar.iesd@bhu.ac.in; 7 Department of Biochemistry, Institute of Science, Hindu University, Varanasi, 221005, India; chetan.keswani4@bhu.ac.in

**Keywords:** abiotic stresses, environmental contaminants, heavy metals, nanoparticles, soil

## Abstract

Abiotic stress in plants is a crucial issue worldwide, especially heavy-metal contaminants, salinity, and drought. These stresses may raise a lot of issues such as the generation of reactive oxygen species, membrane damage, loss of photosynthetic efficiency, etc. that could alter crop growth and developments by affecting biochemical, physiological, and molecular processes, causing a significant loss in productivity. To overcome the impact of these abiotic stressors, many strategies could be considered to support plant growth including the use of nanoparticles (NPs). However, the majority of studies have focused on understanding the toxicity of NPs on aquatic flora and fauna, and relatively less attention has been paid to the topic of the beneficial role of NPs in plants stress response, growth, and development. More scientific attention is required to understand the behavior of NPs on crops under these stress conditions. Therefore, the present work aims to comprehensively review the beneficial roles of NPs in plants under different abiotic stresses, especially heavy metals, salinity, and drought. This review provides deep insights about mechanisms of abiotic stress alleviation in plants under NP application.

## 1. Introduction

In the current scenario, population explosion has emerged as one of the major challenges, especially for sustainable food production, in feeding the growing population [1]. The world population may reach up to 10.9 billion by 2100 and will lead to an increase in demand for food by nearly 50%. To achieve the “Zero Hunger” goal, which is one of the goals of sustainable development of the UN to be achieved by 2030, there is an urgent need for revolutionizing conventional agricultural practices. Such changes can be achieved by employing eco-friendly and sustainable innovations [1,2,3,4].

Plants are unable to move physically from their location to prevent the consequences of environmental stress such as abiotic stresses. Among different abiotic stresses, heavy metal (HM) contamination, soil salinity, and drought stress are described to limit the crop productivity by multiple orders of magnitude [5,6,7]. These changes under abiotic stress trigger perturbations in the metabolism of plants, thereby facilitating reorganization of the metabolic network in order to keep the vital metabolic processes active [8,9,10,11,12].

Soil pollutants, especially HMs and metalloids such as Cr, Cd, Ni, Zn, As, and Hg, are identified as the most commonly detected contaminants [13,14]. The increased release of HMs in the terrestrial environment has been documented to severely affect the productivity of cultivated areas [15,16]. Furthermore, most of the metal contaminants eventually find their way into the terrestrial and aquatic environment, thereby directly or indirectly affecting human health and the associated ecosystems [17,18]. Additionally, there are ample chances of accumulation of HMs in plants exposed to contaminated areas [19].

Salinity and drought stresses are devastating stresses that are reported to limit the economic yield of several crops via inducing biochemical and physiological perturbations [20,21,22]. These stresses confine the plant productivity and growth due to osmotic stress, nutritional imbalance, and oxidative stress [23]. The salt stress results in the accumulation of sodium (Na⁺) and chloride (Cl^−^) ions in the cytosol, eventually causing considerable damage to the cell [24]. Drought stress is known to induce stomatal closure, to inhibit photosynthesis, to reduce the leaf area, to reduce the biomass and growth, to decrease the water potential, to increase the amount of osmolytes, and to induce the generation of reactive oxygen species (ROS) [25]. Abiotic stress triggers perturbation in the metabolism of plants, thereby facilitating reorganization of the metabolic networks in order to keep the vital processes active [8,9,10,11,12]. Thus, the onset of abiotic environmental stressors because of immobile nature of plants eventually leads to reduced crop productivity.

Numerous stress management strategies have been developed by researchers in recent decades. Among them, nanotechnology is one of the emerging strategies that has been anticipated to improve crop productivity [2,3]. Nevertheless, most of the research focusing on nanoparticles (NPs) currently is concentrated on their toxicity [4,26,27,28,29]. Relatively fewer publications are available regarding the role of NPs in crop protection, especially under various abiotic stress conditions [8,30,31].

Nanoparticles may be described as materials with diameters between 1 to 100 nm in at least one dimension [32]. Metal and metal-based NPs show various physiochemical features that are different from their native bulk compounds. The application of NPs has gained widespread popularity in agriculture and allied sectors including various other fields, i.e., the chemical, optical, biomedical, pharmaceutical, food, and textile industries [33,34]. Different NPs for field applications such as nano-agrochemicals have been used to increase agricultural productivity. Some of them include phosphorous NPs (Ca_5_(PO_4_)_3_OH), calcium NPs (CaCO_3_), Mg NPs, ZnO NPs, Fe_2_O_3_ NPs, TiO_2_ NPs, Ag NPs (AgNO_3_), Mn NPs (MnSO_4_), Cu NPs (CuO), Mo NPs, SiO_4_ and AlO_4_ CNTs (carbon nanotubes), and a complex of Chitosan with Zn or Cu [4,35].

Nanoparticles as soil-improving agents, nano-fertilizers, nano-pesticides, growth stimulators, and nano-sensors for controlling various agricultural factors in the farm [36] have been utilized for improving crop yield. It has gained popular acceptance for its potential application in the smart and controlled delivery of pesticides and herbicides and in the sustained-release of fertilizer formulations. Additionally, the contribution of NPs in the alleviation of abiotic stress-induced toxicity in plants is of immense agricultural importance. The intervention of nanotechnology has demonstrated effectiveness not only in the removal of non-degradable metals, but also in the detoxification of slowly degrading contaminants [37]. The past decades have substantially received tremendous contribution about NPs improving plant growth and soil characteristics, particularly in the management of marginal soils affected by HM contamination [38,39]. In a recent study, the contents of chlorophyll (a and b) and carotenoids were noticeably enhanced by magnetic NP treatment to *Hordeum vulgare*, apart from the positive impacts on the genes of photosystems [40]. Likewise, the negative impacts of drought and salinity stress have also been mitigated by the use of NPs [41,42].

In order to obtain in-depth knowledge on field applications of NPs, the present review aimed to discuss the challenges of different abiotic stresses causing substantial changes in crops at the morphological, anatomical, biochemical, and physiological levels and the possible roles and mechanisms of NPs for mitigating the negative consequences of abiotic stresses to improve the agricultural productivity.

## 2. Alleviation of Heavy Metal Toxicity in Plants Using Nanoparticles

Excessive release of HMs in the environment by an exponential rise in anthropogenic activities and industrial processing is of great concern [43,44]. The risks of HM contamination in cultivated fields and aquatic environments due to the indiscriminate addition of various agro-fertilizers are of considerable concern [45,46]. The abundance of HMs in a given environmental matrix beyond certain limits exerts toxicity because of accumulation and genotoxic, carcinogenic, and mutagenic behaviors [46,47,48,49].

The HMs and metalloids associated with the environmental and human health concerns include Cu, Zn, Cd, Cr, Pb, As, and Hg [50,51]. Therefore, it is imperative to develop innovative and economical technologies for the successful elimination of HMs from contaminated sites. However, the characteristic toxicity at low concentrations, slow removal using conventional approaches, and non-biodegradable attributes of HMs [49,52,53,54] are the important factors imposing restrictions in successful detoxification from contaminated sites. The fabrication of effective and eco-friendly NPs for successful employment in managing widespread contamination of hazardous HMs has received much popularity [55]. Among the different metal and non-metal-based NPs, generally, those with an environmentally friendly nature, cost-effectiveness, and ease of availability are preferred for application in environmental clean-up programs as well as in the alleviation of toxicity [56].

The contribution of myriads of NPs in overcoming the challenges of HM-induced toxicity has been presented by various researchers worldwide (Table 1). In general, it has been observed that NPs minimize the uptake of HMs by modifying the expression of genes responsible for metal uptake and by reducing HM bioaccumulation. Furthermore, NP treatment improves the physiological and biochemical parameters of the plants such as enhancing the synthesis of defense enzymes (SOD, POX, CAT, APX, etc.); augmenting nutrient uptake; decreasing the loss of electrolytes; improving pigments and soluble proteins; reducing peroxidation; and causing rise in the levels of proline, glutathione, and phyto-chelatins. These attributes are primarily responsible for the overall increase in the tolerance of the crops and may vary slightly according to different plant species.

Plants employ two strategies to mitigate toxic effects by HMs and to protect their organs, i.e., restricting the uptake and accumulation of HMs with tolerance mechanism applications. The plants accumulate HMs within the plant cell; therefore, remediation is possible by such species. The plants restrict this uptake; thus, farming of such crops is possible without any significant accumulation of HMs in the plant biomass. Noman et al. [57] suggested the contribution of bacterially synthesized Cu NPs in counteracting Cr-induced toxicity in *Triticum aestivum* L. The Cu NPs amended in the soil at the rate of 25 and 50 mg/kg were shown to improve the growth, biomass, and antioxidant pool. The NP treatment was found effective in lowering the synthesis of ROS as well as Cr transport into the plant under Cr stress by substantial soil immobilization. Similar reports on microbially fabricated Cu-based NPs facilitating the alleviation of Cr-induced toxicity and reduced translocation in plant parts conferred by metal immobilization with the resultant improvement in nutrient uptake and biomass were also presented [58]. The mitigation of rising challenges linked with Cd phytotoxicity in *Oryza sativa* L. was recently demonstrated using Fe_2_O_3_ NPs [59]. The addition of NPs improved the level/activity of detoxifying enzymes, photosynthetic potential, and nutrient uptake attributes. Noticeably, the application of NPs helped in reducing the formation of ROS, lowered the expression of genes supporting the transport of Cd, and restricted mobilization in leaves. For example, the application of Fe_2_O_3_ NPs reduced Cd transport by 70% in *T. aestivum* L. [60].

Researchers have suggested the application of biologically synthesized NPs for the management of HM-contaminated agricultural lands and the improvement of plant growth and development [61]. The foliar application of TiO_2_ in counteracting the effects of Cd in *Zea mays* as opposed to root supplementation was observed [62]. Treatment with different sizes of Si NPs led to a reduction in growth inhibition of *Glycine max* caused by Hg [63]. Moreover, Si NP-assisted improvement in chlorophyll content as declined by Hg exposure was also observed. The amendment of Si NPs led to a lowered accumulation of Hg in roots and shoots as confirmed by X-ray fluorescence and was proposed as an innovative methodology to passivate volatile contaminants such as Hg.

Recently, the investigation on the effectiveness of Au NPs to alleviate the hazardous effects induced by Cd in *O. sativa* L. under hydroponic conditions was presented [64]. The treatment of *O. sativa* L. plantations with Au NPs caused considerable reductions in the level of Cd in roots and leaves by 33 and 46.2%, respectively. Media supplementation with the NPs in question caused an improvement in antioxidant defense enzymes facilitating the diminution of oxidative stress in *O. sativa* L. challenged by Cd. The application of Au NPs also helped in restricting the expression of genes closely associated with metal transport across the cells [64]. Similar investigations on the role of different NPs such as Si NPs [65], biologically synthesized Cu NPs [58], ZnO NPs [66], Ti NPs [67], Se NPs [68], and Fe_2_O_3_ NPs [60] in metal detoxification in different plant parts were also reported.

Research conducted by different investigators suggests the amelioration of toxic effects of HMs by NPs via improvements in antioxidant defense enzymes, modifications in the expression of metal transporter genes, altered regulation of vital metabolic pathways, variation in metal accumulation/mobilization, maintenance of photosynthetic pigments, and reduction in growth inhibition under metal stress [19,64,67,69].

## 3. Alleviation of Salinity Stress in Plant Using Nanoparticles

**Figure 1:**
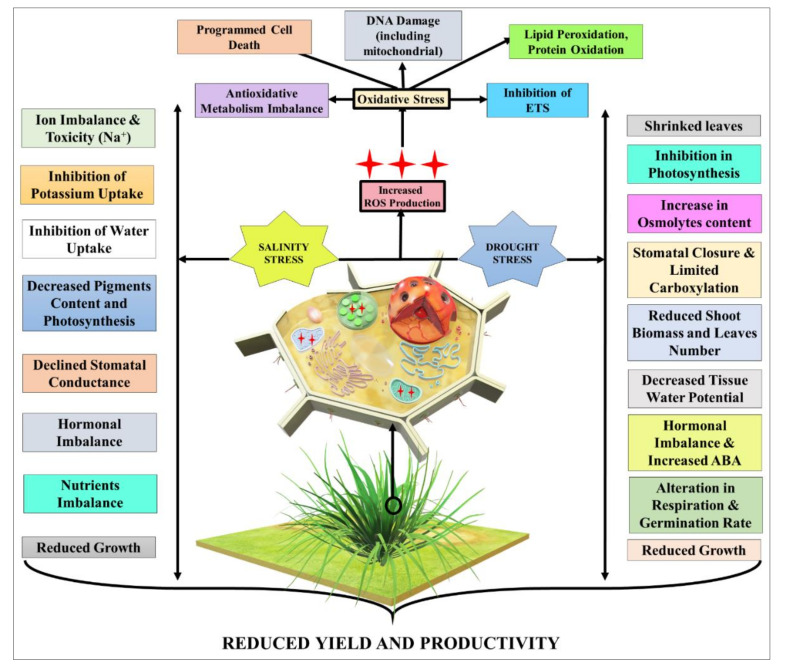
Salinity and drought stress-mediated responses in the plants; ETS: electron transport system.

Salinity has emerged as a global concern due to steady increases in salt-affected land throughout the world [76]. For example, it is straddling from the Indo-Gangetic plain to the Great Hungarian Plain, Russia, Israel, China, and the United States of America [77,78]. The extent of salinity-affected areas is expected to cover about 50% of total agricultural land by 2050. Salinity stress causes various detrimental effects to plants’ physiological, biochemical, and molecular features and reduces productivity [79]. These impacts and their consequences induced by salinity stress in plants are shown in Figure 1.

Nanoparticles can help the plant under salt stress by regulating ion balance; reducing the Na^+^ ion toxicity; increasing the uptake of K^+^; activating the antioxidative defense system; increasing the contents of the pigment, compatible solutes; and increasing stomatal conductance. In salinity-stressed *T. aestivum* L., the application of magnetite NPs improved chlorophyll contents and antioxidative enzymes along with the amelioration of various polypeptide chains, which are reported to be linked with salinity stress tolerance [80]. Nano-SiO_2_ improved the growth of *G. max* under salt stress by raising the level of leaf K^+^ and biological antioxidant activities [81]. Similarly, in salt-stressed *T. aestivum* L. cultivars, nano-SiO_2_ was found to improve seed germination and growth [82].

The application of Zn NPs to salt-stressed *Brassica napus* plants alleviated the salinity-induced detrimental impacts by upregulating the antioxidative mechanism, osmolyte biosynthesis, and ionic control [83]. In *Solanum lycopersicum*, Cu NPs applied to the leaves mitigated salinity stress by improving growth and the Na^+^/K^+^ ratio. Moreover, Cu NPs improved the level of glutathione, polyphenols, and vitamin C content as compared to the control. Additionally, the activities of APX, GPX, and SOD were also modulated, thereby improving the overall plant’s normal growth and development [84]. In addition, seed priming with ZnO NPs (60 mg/L) ameliorated the detrimental consequence induced by the NaCl treatment in *Lupinus termis* via increasing the pigments, osmoregulation, and regulation of the contents of stress-associated metabolites. In another study, the seed priming of *T. aestivum* L. with Ag NPs was also proven to be an adequate salinity stress management strategy [85].

Recently, a study depicted that the exogenous application of salicylic acid+nano-Fe_2_O_3_ to *Trachyspermum ammi* L. alleviated salinity stress to a considerable extent via increasing K^+^ uptake, K^+^/Na^+^ ratio; iron content; activities of various antioxidative enzymes *viz*. SOD, catalase (CAT), peroxidase (POD), and phenol peroxidase (PPO); and the contents of the compatible solutes. These modifications collectively led to the improvement in membrane stability index, leaf water content, pigments, and growth of the plants (Table 2) [86].

## 4. Alleviation of Drought Stress in Plants Using Nanoparticles

Drought stress is reported to have severe consequences for crops, including reduced leaf area, reduced growth, limited carboxylation, decreased water potential, hormonal imbalance, and oxidative stress [95,96]. It is frequently associated with high temperature due to increased water loss through evapotranspiration. Owing to this reason, plants reduce the leaf water and turgor pressure. These consequences are also associated with the stomatal closure, which in turn decelerates the plant’s metabolism and ceases vital enzymatic reactions. In addition, the severe water shortages eventually contribute to stunted crop growth and finally death [97,98].

An insight into the morphophysiological responses and consequences of plants against drought stress is presented in Figure 1. The key factors under drought stress are the severity and duration of the stress, which could be directly correlated with drought stress-induced loss in crop productivity and economic yield [99]. Furthermore, salinity combined with drought stress led to a decrease in water potential, but the osmotic potential decreased more significantly [100].

Applications of NPs to drought stress plants have been observed to improve photosynthetic rate, stomatal conductance, relative water content, and ameliorated cell membrane damage by lowering the contents of stress metabolites and electrolyte leakage. Furthermore, increases in osmolyte contents, carotenoid content, chlorophyll content, protein content, and phenolic substances (i.e., rosmarinic acid and chlorogenic acid) and improved activities of antioxidant enzymes such as CAT, SOD, and POX have also been found as a general mechanism in overcoming and mitigating drought stress.

The experimental outcomes of Ashkavand et al. [101] revealed the positive impacts of Si NPs on physiological parameters under drought stress. In another study, the application of nanoscale TiO_2_ improved the morphophysiological indices, especially at lower doses under water deficit environment, which subsequently improved the plant’s performance [102]. The application of TiO_2_ NPs in *Dracocephalum moldavicum* also upsurged the synthesis of some important metabolites such as rosmarinic acid, chlorogenic acid, acacetin-7-*O*-glucoside, and apigenin-7-*O*-glucoside [103]. Zinc oxide NPs were also reported as potential novel fertilizers that can provide new horizons for the agricultural sector. The findings reported by Dimkpa et al. [104] indicated that Zn in nanoparticulate form can hasten the phenological development, reproductive yield, and nutrition in water-stressed cereal crops.

According to the research outcomes of Zahedi et al. [105], in drought-stressed *Fragaria ananassa*, the chlorophyll contents were improved after spraying Se and SiO_2_ NPs. The applications of these NPs also resulted in significant improvement in other physiological parameters such as MDA, H_2_O_2_, and antioxidative enzymes activities. Similarly, zero-valent Cu NPs showed the ameliorative roles against drought stress in *Z. mays*. The drought stress tolerance was imparted by maintaining the leaf water content; increasing the anthocyanin, chlorophyll, and carotenoids contents; and enhancing the ROS scavenging potential [106].

Moreover, the exogenous exposure of ZnO NPs to drought stressed *Solanum melongena* caused an increase in relative water content and membrane stability index that was observed to be related with the improved stem, leaf anatomical features (vascular cylinder thickness, dimensions of the pith, cortex thickness, and leaf blade), and plant’s photosynthetic efficiency [107]. Therefore, an extensive literature survey was also performed to explicate the positive influences of NPs on edible plants that are grown under drought stressed environment (Table 3).

## 5. Mechanism of Stress Alleviation in Plants under Nanoparticles Application

This section investigates the influence of NPs on enzymatic and non-enzymatic components and how the interaction of NPs with these components helps the plant in coping with stress conditions. This section also discusses the impacts of NPs on a plant’s secondary metabolism, the activation of the salt overly sensitive (SOS) pathway, the roles of abscisic acid under salt stress, and molecular insights.

### 5.1. Impact on Antioxidative Enzymes

During oxidative stress, SOD helps in the removal of superoxide (•O_2_^−^) by catalyzing dismutation reaction and by converting it into O_2_ and H_2_O_2_. The CAT, POX, and GPX metabolize and carry out the conversion of H_2_O_2_ into water and O_2_. Together, SOD, CAT, POX, and GPX prevent the formation of more harmful ROS (such as hydroxyl radicals) via a Haber Weiss reaction. Furthermore, the Foyer–Halliwell–Asada pathway (also known as the ascorbate-glutathione pathway), which involves enzymes such as glutathione reductase (GR), APX, monodehydroascorbate reductase (MDHAR), and dehydroascorbate reductase (DHAR), also reduce H_2_O_2_ after utilizing the reducing potential of NADPH (Table 4).

The SOD enzyme is present in three different isoforms in plants. i.e., Cu/Zn-SOD (in cytoplasm, peroxisomes, chloroplasts, and apoplasts), Fe-SOD (in chloroplasts), and Mn-SOD (in mitochondria and peroxisomes) [8]. In an experiment, it was revealed that, when Cu NPs were applied to hydroponically grown *Cucumis sativus*, the gene expression level of Cu/Zn-SOD increased by up to six-fold under 50 mg/L Cu NPs treatment [111]. In this study, the biomass of *C. sativus* was found to be reduced under NPs treatment and plant was found under stress conditions, leading to increased activity of the SOD [111]. However, in another report, it was observed that the treatment of ZnO NPs decreased the activity of SOD in *Cicer arietinum* [112]. In this study, ZnO NPs were found to be beneficial for *C. arietinum* growth and reduced ROS levels [112]. Therefore, the differences in the change in the activity of SOD in (*C. sativus* and *C. arietinum*) were due to the different responses of the plant to these NPs.

In another study, the beneficial role of ZnO NPs was observed in comparison to the bulk ZnO for three different vegetable crops, i.e., *B. oleracea var. capitata*, *B. oleracea var. botrytis*, and *S. lycopersicum* [113]. It was found that treatment with ZnO NPs increased the germination, growth, and biochemical parameters of these crops in comparison to the bulk ZnO. The ZnO NPs were found to induce the activity of antioxidant enzymes such as SOD, CAT, APX, and guaiacol peroxidase (POD) along with the pigments, sugar, and protein contents in these three crops. However, the activities of these enzymes were even higher under bulk ZnO, indicating that the activities of these enzymes are influenced by physiological state and oxidative stress within plant cells, and bulk ZnO was found to exert more stress in these horticultural crops. These findings suggest that changes in the activity and gene expression level of SOD were influenced by the overall biochemical state of the plant cell rather than metallic NPs directly influencing SOD activity. However, more evidence is required to support the hypothesis. In another report, it was revealed that, when Se NPs and Cu NPs were applied on *S. lycopersicum*, it resulted in an increased yield; better contents of chlorophyll, vitamin C, and glutathione; and increased activity of SOD, GPX, and phenylalanine ammonia-lyase (PAL) [114].

Thus, in this study, the physiological parameter showed a better state of the health condition in the plant cell. In a similar study, ZnO NPs were applied to *Leucaena leucocephala* along with phosphorus [70]. In this study, plant biomass, chlorophyll, carotenoid, and protein content were found to be increased significantly. In contrast, the activities of SOD and POX were also found to be high.

However, the exploration of how the antioxidant enzymes are relatively more active during such NP treatments is required. However, it is still uncertain whether the applications of Se NPs, Cu NPs, or ZnO NPs have direct effects on the activities of these enzymes or whether the activities of these enzymes under NPs stress are accountable for the plant’s improved physiological condition and yield. Therefore, more investigations are required to fully comprehend the mechanisms behind the interaction of the NPs with these antioxidant enzymes.

In one study, when Cu-chitosan NPs were applied to boost defense responses against *Curvularia* leaf spot (CLS) disease in *Z. mays*, it was observed that application of NPs induced the activity of antioxidant enzymes (SOD and POD) and defense enzymes such as PAL and polyphenol oxidase (PPO) [115]. This study highlights the application of Cu-chitosan NP-induced innate immunity by the elicitation of the plant defense and antioxidant system for disease protection, thereby promoting plant growth [115]. Similarly, ZnO NPs coated with phytomolecules were also found to promote the growth (by 125.4%), biomass (by 132.8%), pigment, and protein content of the *Gossypium hirsutum* plants [116]. Furthermore, this study reported that there was a significant increase in the activities of SOD (267.8%) and POX (174.5%) but a decrease in the activity of catalase (CAT, 83.2%) in the NP-treated plants as compared to the control [117]. These findings suggest that ZnO NPs coated with biomolecules can have a significant positive impact on the health of the plant cell and can boost the immunity of the plant by increasing the activity of the antioxidant enzymes.

The nanoform of a micronutrient acts as an elicitor in activating antioxidant enzymes and therefore increases the tolerance capacity of the plant to various kinds of stress conditions. To prove this, an experiment was conducted, where the nanoform of TiO_2_ was compared to its bulk form to understand the impact on antioxidant enzymes in *Hyoscyamus niger* [118]. It was observed that both SOD and POX enzyme activities were higher in nanoform-treated plants than that of the bulk form, except CAT activity. [118]. Therefore, it must be thoroughly studied further to clearly understand the influence of the NPs with reference to antioxidant enzymes.

### 5.2. Impact on Cellular Osmolyte Pool

The role of the non-enzymatic component has also been investigated under the influence of NP treatment. The activity of the antioxidant enzymes was correlated with the proline content, which acts as a non-enzymatic defense molecule in the plant cell. The growth, photosynthetic efficiency, and antioxidant system increased along with proline content in *S. lycopersicum* treated with ZnO NPs. Thus, the improved antioxidant defense system and proline accumulation could provide stability to *S. lycopersicum* [119]. The biochemical responses of *S. lycopersicum* grown under salt stress were investigated after the foliar application of Cu NPs, and it was revealed that oxidative damage due to salt stress can be mitigated by application of Cu NPs. It was observed that the activity of enzymatic (APX, GPX, SOD, and CAT) and non-enzymatic components (phenol, vitamin C, and glutathione) both helped in increasing the stress tolerance in *S. lycopersicum* [84].

The phytotoxicity of Al in acidic soil was ameliorated after the application of nano-SiO_2_ in *Z. mays* [120]. It is important to note that NP treatment did not influence Al accumulation; rather, it induced the enzymatic (SOD, CAT, APX, GPX, and GST) and non-enzymatic (ascorbate, glutathione, polyphenols, flavonoids, and tocopherols) defense systems of the plant to mitigate Al toxicity [120]. Moradbeygi [121] investigated the impact of FeO NPs on *Dracocephalum moldavica* growing under salinity stress. It was observed that, due to salinity stress, plants exhibited a decrease in leaf area. However, after the application of FeO NPs, plants were able to tolerate salinity stress due to the increased activity of antioxidant defense enzymes as well as an increase in the concentration of non-enzymatic components. Total phenolics, flavonoids, and anthocyanin content as well as the activity of the guaiacol peroxidase (GOPX), APX, CAT, and GR enzymes were enhanced. The application of FeO NPs significantly increased the leaf area in the plants under salt stress [121]. Similarly, in another study, nano-silicon when applied to *G. max* under salt stress led to increases in CAT, POX, APX, and SOD enzyme activities along with increases in concentration of phenolic components, ascorbic acid, and α-tocopherol contents, thereby improving the salt tolerance [122].

**Figure 2:**
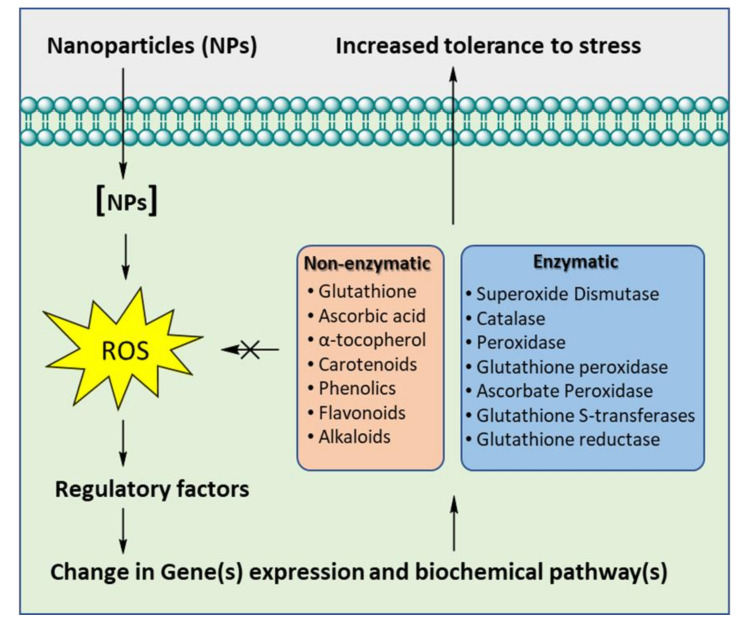
Schematic representation of elicitation of the enzymatic and non-enzymatic antioxidant defense mechanisms of plants by nanoparticles.

These studies demonstrated that the use of nano fertilizers can be an effective strategy to overcome various abiotic stresses in the plants. Many of the non-enzymatic components also serve as high value compounds (such as flavonoids, phenolics acids, alkaloids, and carotenoids) for various downstream applications. The potential of NPs treatment in eliciting the production of these bioactive compound have been reviewed recently by Rivero-Montejo et al. [123]. These studies comprehensively signify the role of non-enzymatic components of the antioxidant defense system of the plants under NP influence (Figure 2).

### 5.3. Molecular Insights

The miRNAs are important in almost all aspects of normal plant growth and production as well as in response to environmental variations such as light, nutrients, and various abiotic and biotic stresses [124]. In this context, the interaction of NPs with miRNAs was also recorded to alleviate several abiotic stress-induced consequences. For instance, the treatment of TiO_2_ NPs at low doses to tobacco plants caused the upregulation of miRNA under metal stress; however, increased levels of NPs caused wilting, reduced biomass, leaf sizes, and leaf numbers as well as decreased root development [125]. The transcriptional responses of plants (*Arabidopsis* sp.) treated with Ag NPs were recorded using whole genome cDNA expression microarrays. The outcome of this work revealed that there was the upregulation of several genes (286), which are commonly reported to be associated with metal and oxidative stress [126]. It is documented that the NP-mediated benefits for plants relied on their association and absorption, which leads to several changes at the molecular level that ultimately influences the plant’s physiology [127].

The impacts of Ce NPs on *Gossypium* under salinity stress were investigated in terms of the morphophysiological, biochemical, and molecular pathways. The research outcomes of this study revealed that there was an increase in biomass and development as well as a differential root transcripts expression in response to seed priming with Ce NPs [128]. However, there are only few papers that describe the plant–NP interaction at the molecular level; therefore, there is a great scope of research in this area for the application of NPs in the agricultural sector by modulating the molecular aspects of plants. According to a study, new plant genetics approaches based on NP-mediated clustered regularly interspersed palindromic repeats (CRISPR)-associated protein technology, similar to those in other biological systems, can deliver ground-breaking innovation.

### 5.4. Impact on Secondary Metabolites

Stimulation in the secondary metabolites (terpenoids, phenolic compounds, alkaloids, etc.) under NPs treatment has been observed as one of the abiotic stress mitigation strategies. The levels of some phenolic compounds were increased in *Hordeum vulgare* after the exposure of CdO NPs for 3 weeks [129]. Similarly, the application of ZnO NPs increased the total phenolics and anthocyanin content in the *Solanum tuberosum* [130]. Likewise, ZnO NPs were found to induce the production of secondary metabolites, especially flavonoid contents in *Echinacea purpurea* [131]. Such an increased flavonoid content in the extract of *Echinacea purpurea* was found to exert anticancer properties. The NPs can be considered as elicitors for the production of secondary metabolites, and this potential has been explored by Modarresi et al. [132]. In this study, a significant increase in the total flavonoid and total alkaloid content (particularly glaucine, quercetin, and kaempferol) was observed in *Nigella arvensis* plants after treatment with different NPs such as TiO_2_, Al_2_O_3_, and NiO [132]. However, more investigations are required to understand if such an increase in anthocyanin, flavonoid, and alkaloid contents under NP influence has a beneficial role for the plant to cope with the abiotic stress conditions.

The application of nano-ZnO (100 mg/L) in *Z. mays* enhanced the synthesis of melatonin and activated the antioxidative enzymes, which alleviated drought-induced consequences. These benefits related with the upregulation of SODs, APX, CAT, TDC (tryptophan decarboxylase), T5H (tryptamine 5-hydroxylase), SNAT (serotonin N-acetyltransferase), N-acetyl serotonin O-methyltransferase (ASMT), and COMT (caffeic acid O-methyltransferase) [133]. Likewise, the drought-stressed *Punica granatum* treated with Se NPs by foliar spraying showed higher levels of pigments, phenolic content, osmolytes, antioxidant enzymes, and abscisic acid than untreated ones [134].

### 5.5. Activation of Salt Overly Sensitive (SOS) Pathway and Roles of Abscisic Acid under Salt Stress

**Figure 3:**
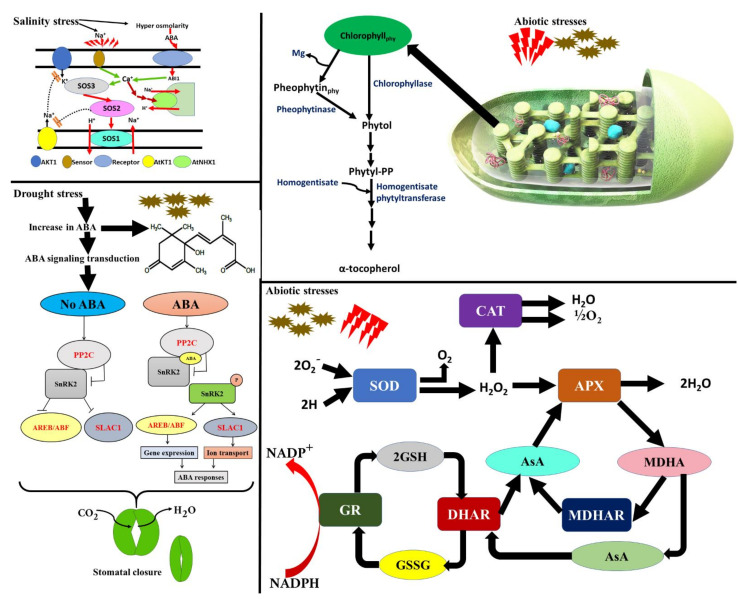
Schematic presentation of the underlying pathways that regulates the plant’s responses under a stressed environment.

There are several studies that have demonstrated the significant roles of the SOS pathway in the maintenance of ionic homeostasis at the cellular level and salt tolerance [135]. Briefly, the SOS signaling pathway consists of three main proteins, namely, SOS1, SOR2, and SOS3 (Figure 3). The SOS1 gene encodes a plasma membrane antiporter (Na^+^/H^+^) that regulates Na^+^ efflux along with the distant transport of Na^+^ from the roots [136]. It is further characterized by a long C-terminal that comprises the putative nucleotide-binding motif plus autoinhibitory domain. The SOS2 is reported to encode a serine/threonine kinase that is activated by elicited Ca^+^ signals induced by salt stress [137]. The C-terminal regulatory domain of SOS2 protein contains a FISL motif. The third component of the SOS pathway is SOS3 protein that is a myristoylated Ca^+^ binding protein and possesses a myristoylation site at its N-terminus (important for salt tolerance). This motif acts as a site of interaction for Ca^+^ binding of SOS3 protein. The interaction of SOS2 and SOS3 proteins results in the activation of kinases that phosphorylates SOS1. Under salinity stress, the increase in Na^+^ leads to a sharp increase in Ca^+^ [138].

Applications of NPs may activate the SOS signaling pathway, which is a crucial mechanism for Na^+^ exclusion and ionic homeostasis in salinity-stressed plants, whilst the information is too scarce to reach definite conclusions in this milieu. For instance, the supplementation of multi-walled carbon nanotubes (MWCNTs) with the treatment of carbon nanotube in *B. oleracea var. italica* under salt stress conditions resulted in alterations in root plasmalemma permeability as well as improved aquaporin transduction [139]. In another study, the application of MWCNTs was observed to induce modulation in the genes of SOS1 in salt-stressed plants of *B. napus* L. [140]. Contrarily, other mechanisms are also observed to operate in blocking the bypass route of Na^+^ in salinity-stressed plants by the treatment as a protective strategy. The research outcomes of some studies demonstrated the roles of Si in the obstruction of Na^+^ uptake by silicate polymerization in the exodermis and endodermis, enhanced lignification along with suberization, and Casparian band’s formation [141,142,143].

Abscisic acid (ABA) is characterized as a stress hormone, accumulates in response to stress, and mediates numerous stress responses to acclimatize the plants accordingly. It is well reported to participate in water-limited environments that can be the result of drought and salinity stresses. ABA is essential to the regulation of water deficit in plants and to the induction of genes by such enzymes and other proteins that are tolerant to cell dehydration [144].

In the ABA-dependent pathway, it binds with its receptors, i.e., Pyrabactin Resistance Protein1/PYR-like Proteins (RCAR/PYR1/PYLs), and belongs to the START-domain superfamily of proteins [145]. In plants, receptors of RCAR/PYR/PYL are reported to be in the cytoplasm and nucleus. As this hormone binds to RCAR/PYR/PYLs, the inactivation of type 2C protein phosphatases (PP2Cs) takes place. This subsequently causes the suppression of PP2C-mediated dephosphorylation of Sucrose nonfermenting Kinase-1-Related protein kinase 2s (SnRK2s) [95].

Thus, an activated SnRK2s switches the ABA-dependent gene expression and ion channels that help the plants cope with the stress. On the other hand, in ABA-independent pathway, PP2Cs are observed to dephosphorylate SnRK2s inhibiting the kinase activity, which prevents downstream gene expression [144,145].

The ABA was entrapped in monodispersed mesoporous silica (MSN) NPs (diameters ≈20 nm; pore sizes ≈2.87 nm) in which pore the entrances of MSNs were covered with decanethiol gatekeepers via GSH-cleavable disulfide linkages. In this work, the treatment of MSNs with redox-responsive gatekeepers was applied to drought-stressed *Arabidopsis* seedlings. The results showed that the usage of ABA in this controllable way can provide stress mitigation benefits [146].

## 6. Conclusions and Perspectives

It is demonstrated that NPs can act as a potential stimulating agent, especially for germination, growth, and yield of various plants. Additionally, their applications can protect plants from detrimental consequences induced by numerous abiotic stresses because NPs display a moderately wide spectrum of actions (increase in water uptake into seeds, metabolism of starch reserves, stimulation of photosynthesis, changes in phytohormone levels, modulation of oxidative stress, or influenced uptake of nutrients) [65,155]. However, the majority of the studies have been conducted to understand one or other types of stress conditions. Future studies need to focus on more realistic stress condition in a practical scenario. The beneficial role of NPs on plant health has been proven by many studies; however, the exact understanding of the molecular mechanism behind the increased tolerance of plants is not clearly understood yet. Therefore, more investigation is required to understand how the NPs influence the antioxidant system of the plant cell, leading to increased tolerance to various kinds of stress. Such an understanding may help in designing smart NPs of the future that can help in mitigating stress and can ensure sustainable agriculture production.

Future studies need to focus on the interaction of NPs with signaling mechanisms leading to a change in gene expression of defense enzymes. In addition to that, currently, there are some limitations in using expensive NPs for agriculture. Treatment with gold and silver NPs is not economically feasible. Therefore, understanding the mechanism behind the workings of these NPs in modulating plant immunity may help in exploring an alternative method of increasing plant tolerance. The field application of many of the fabricated NPs is still very much limited because of changing environmental conditions, soil types, plants to be treated, and most importantly the physicochemical nature of metallic/non-metallic NPs. The constraints associated with field applications include the toxicity and accumulation of NPs in crop plants. Future studies pertaining to the appraisal of toxicological effects on model microbes, flora, and fauna are crucial to implementing nanotechnology for field application.

Several studies have envisaged the beneficial roles of NPs in the amelioration of salinity, heavy metal, and drought stresses. Nevertheless, the synergistic effect of the different treatments still needs further investigations to make concrete conclusions. Moreover, long-term studies on cereal crops and other strategic crops are suggested to demonstrate the relationship between NPs dose, soil types, and ecological impacts. Caution is necessary when evaluating the toxicological impact of NPs on different organisms and environments before utilizing their potential benefits.

## Figures and Tables

**Table 1:** Applications of NPs in the mitigation of HMs stress by altering the morphophysiological responses of plants.

Nanoparticles	Plants	Germination and Morphological Responses	Physiological Responses	References
Si (10 μM)	*Pisum sativum* L.	Presence of Si NPs improved the growth in presence of Cr	Si NPs minimized the Cr storage, enhanced the synthesis of defense enzymes and augmented nutrient uptake	[69]
ZnO (25 mg/L)	*Leucaena leucocephala*	Application of NPs induced seedling growth	ZnO NPs amendment improved pigments and soluble proteins, reduced peroxidation; there was rise in the antioxidant defense enzymes	[70]
Fe_3_O_4_	*Triticum aestivum* L.	Fe_3_O_4_ NP treatment minimized the inhibitory action of HMs	Fe_3_O_4_ NPs supplementation improved the level of superoxide dismutase and peroxidase	[71]
Si (19, 48, and 202 nm)	*Oryza sativa* L.	Si NPs enhanced the number of cultured cells and decreased proportionally with the rise in NP size; the treatment maintained the cellular integrity in the presence of metals	Si NPs amendment caused altered expression of genes responsible for reduced metal uptake	[72]
ZnO (0, 50, 75, and 100 mg/L)	*Zea mays* L.	Treatment caused rise in plant length, leaf number, and biomass	ZnO NPs application enhanced chlorophyll content, gas exchange characteristics, and antioxidant enzymes; addition led to reduced content of Cd in root and shoot	[73]
ZnO (0, 25, 50, 75, and 100 mg/L) and Fe NPs (0, 5, 10, 15, and 20 mg/L)	*T. aestivum* L.	Treatment induced plant growth, dry weight, and grains under Cd stress	Addition of NPs decreased the loss of electrolyte and activity of superoxide dismutase and peroxidase along with diminished Cd accumulation	[74]
Si	*Glycine max* L.	Si NPs minimized the growth inhibitory action of Hg	Incorporation of Si NPs improved the chlorophyll content and reduced the Hg content in root and shoot	[63]
Mel-Au (200 μM)	*O. sativa* L.	—	Application of Mel-Au NPs caused reduction of Cd level in root and shoot, improved chlorophyll content and raised the activity of antioxidant enzymes	[64]
Fe (25 and 50 mg/L)	*O. sativa* L.	Treatment of Fe NPs improved plant length and dry weight	Fe NPs application caused rise in the level of proline, glutathione and phyto-chelatins; Fe NPs addition led to improved defense enzymes and glyoxalase machinery	[19]
ZnO (10–100 mg/L)	*O. sativa* L.	Amendment of ZnO increased the growth of seedlings	Treatment facilitated reduced accumulation of arsenic in root and shoot together with rise in phytochelatin level	[75]
Cu (25, 50, and 100 mg kg^−1^ of soil)	*T. aestivum* L.	Rise in plant height and shoot dry weight	Increase in N and P content; reduced Cd transport, rise in the level of vital ions and antioxidant pool	[58]
Cu (0, 25, 50, and 100 mg kg^−1^ of soil)	*T. aestivum* L.	Improved biomass and growth	Reduced Cr availability; increase in nutrient uptake; rise in antioxidant content	[57]
Fe_2_O_3_ (0, 25, 50, and 100 mg kg^−1^ soil)	*O. sativa* L.	Improved fresh and dry biomass; increased height	Augmented detoxifying enzymes, photosynthetic potential, and nutrient uptake attributes; reduced formation of ROS, lowered expression of genes supporting the transport of Cd; restricted Cd mobilization in upper plant parts	[59]
Fe_2_O_3_ (25, 50, and 100 mg kg^−1^ soil)	*T. aestivum* L.	Rise in plant fresh and dry biomass; increase in plant length	Reduced Cd transport; enhanced N, P, and K content; increased antioxidants and pigment content	[60]
TiO_2_ (0, 100, and 250 mg/L soil)	*Z. mays*	Foliar application improved shoot and root dry weight	Reduced accumulation of Cd; increased activities of antioxidant enzymes	[61]
SiO_2_ (30 and 50 nm)	*G. max*	Improved seedling fresh weight	Improved chlorophyll content; lowered accumulation of Hg in root	[63]
Au (200 μM)	*O. sativa L.*	—	Reduced level of Cd in root and leaves by 33 and 46.2%, respectively; improvement in antioxidant defense enzyme; restricted expression of genes associated with metal transport	[64]
Si (0, 25, 50, and 100 mg/kg soil)	*T. aestivum L*.	Improved plant height	Improved chlorophyll; photosynthesis; diminished Cd content in tissues;	[64]
ZnO (0, 50, and 100 mg L^−1^)	*G. max*	Improved root and shoot growth	Reduced arsenic concentration in root and shoot; improved photosynthesis, water loss, photochemical yield; raised antioxidative defense enzymes	[66]
Ti (0.1 to 0.25%)	*Vigna radiata L*.	Augmented radicle length and biomass	Decline in the level of ROS and lipid peroxidation; upregulation of genes related with antioxidative enzymes	[67]
Se and Si (5, 10, and 20 mg L^−1^)	*O. sativa L.*	—	Lowered accrual of Cd and Pb; improved yield	[68]

**Table 2:** Applications of NPs in salinity stress mitigation by altering the morphophysiological responses of plants.

Nanoparticles	Plants	Germination and Morphological Responses	Physiological Responses	References
Ag (0, 2, 5, and 10 mM)	*Triticum aestivum* L.	Seed priming with Ag NPs significantly augmented the fresh and dry biomass of salinity stressed wheat plants at all doses compared to the control.	Ag NPs increased the activities of vital antioxidative enzymes whilst declined the contents of stress indicators, i.e., MDA and H_2_O_2_ in wheat leaves as compared to salt stressed plants.	[85]
Zn-, B-, Si-, and Zeolite NPs	*Solanum tuberosum* L., Diamont cultivar	Application of individual and binary treatment of NPs improved plant height, shoot dry weight, number of stems per plant, and tuber yield as compared to the control.	NP treatment increased leaf relative water content, leaf photosynthetic rate, leaf stomatal conductance, and chlorophyll content in comparison to the control; improved nutrients contents, leaf proline content, and leaf gibberellic acid level; and enhanced the contents of protein adn carbohydrates, and antioxidative enzymes’ activities.	[87]
Fe (0, 0.08, and 0.8 ppm), and potassium silicate (0, 1, and 2 mM)	*Vitis vinifera*	—	Application of NPs significantly increased the total protein content, activities of antioxidative enzymes (POD, CAT, and SOD), and hydrogen peroxide, while reduced proline content.	[88]
Fe (0.0, 0.08, and 0.8 ppm)	*Fragaria ananassa*	Application of Fe NPs (at higher concentrations) increased root dry weight and dry weight of the explants.	Fe NPs improved the contents of photosynthetic pigments and total soluble carbohydrate, membrane stability index, and relative water content of salinity-stressed plants.	[89]
N–Na_2_SiO_3_ (400 ppm)	*S. tuberosum* L.	Foliar spraying of N–Na_2_SiO_3_ restored the tuber number per plant and tuber yield along with improved water use efficiency and tuber dry matter percentage under salinity stress.	Application of N–Na_2_SiO_3_ exerted positive impacts on the quantum yield of PS II, carotenoids content, and DPPH radical scavenging activity in salinity stressed plants.	[90]
SiO_2_ (0, 50, 100, and 150 mg/L)	*Musa acuminata*	All doses of SiO_2_ NPs improved the number of shoots and shoot length of banana.	Application of SiO_2_ NPs increased chlorophyll content, lowered electrolyte leakage, reduced MDA content, and altered the content of phenolic compounds	[91]
CNPs (0.3% and 90–110 nm)	*Lactuca sativa*	The salinity-induced deleterious effects on germination and associated parameters were alleviated by the exposure of C NPs, e.g., treatment of C NPs for 2 h significantly improved the germination rate in some varieties.	—	[92]
ZnO (0, 1000, and 3000 ppm)	*Trigonella* *foenum-graecum*	—	Interaction of NaCl and ZnO was recorded to reverse the salinity induced consequences (L-proline, protein, MDA, aldehydes, sugars, H_2_O_2_, and antioxidative enzymes) in both cultivars, but the results were more apparent in case cv. Ardestanian than cv. Mashhadian.	[93]
ZnO (10, 50, and 100 mg/L)	*Lycopersicon esculentum*	Foliar spraying of ZnO NPs increased shoot length and root length, biomass, and leaf area.	Increased chlorophyll content and photosynthetic attributes, protein content, and activities of antioxidative enzymes (POX, SOD, and CAT) in salinity-stressed tomato plants.	[94]
TiO_2_, (40, 60, and 80 ppm)	*Zea mays* L.	Seed priming with TiO_2_ positively impacted the germination (germination percentage, germination energy, and seedling vigor index) and seedling growth (lengths of root and shoot, fresh, and dry weight) and reduced the mean emergence time.	Results showed the enhancement in potassium ion concentration, relative water content, contents of total phenolic and proline contents; increased SOD, CAT, and PAL activities; and decreased sodium ion concentration, membrane electrolyte leakage, and MDA content.	[53]

**Table 3:** Applications of nanoparticles in drought stress mitigation by altering the morphophysiological responses of plants.

Nanoparticles	Plants	Germination and Morphological Responses	Physiological Responses	References
Silica (0, 10, 50, and 100 mg/L)		S NPs increased plant biomass and xylem water potential in drought-stressed seedlings.	S NPs improved the photosynthetic rate and stomatal conductance considerably; no effects were recorded on MDA content, relative water content, and electrolyte leakage index; carbohydrate and proline content under drought stress declined.	[101]
TiO_2_ (0, 10, 100, and 500 mg/L, and 10–25 nm)	*Linum usitatissimum* L.	Number of capsules per plant increased in plants under the application of TiO_2_ as compared to control and enhanced the seed weight.	Nano-TiO_2_ treatment of drought-stressed plants surged carotenoids content and ameliorated cell membrane damage, and seed oil and protein contents (at 100 mg/L) compared to normal.	[102]
Chitosan (0, 30, 60, and 90 ppm)	*Triticum aestivum* L.	Foliar spraying of C NPs (particularly at 90 ppm) at tillering, stem elongation, and heading stages caused increase in leaf area, crop yield, and biomass as compared to the control.	Application of C NPs increased relative water content, chlorophyll content, photosynthetic rate, and CAT and SOD activities in comparison to the control.	[108]
TiO_2_ (0, 5, 10, 20, 30, 50, 100, and 150 ppm)	*Dracocephalum moldavica* L.	TiO_2_ treatment had no significant impact on the plant dry weight	TiO_2_ application (30–50 ppm) increased certain beneficial phenolic substances (rosmarinic acid and chlorogenic acid) in stressed plants.	[103]
Si (0, 25, 50, and 100 mg/kg)	*T. aestivum* L.	Treatment of Si NPs to drought-stressed plant grown in Cd contaminated soil showed maximum values of shoot, root, and grain dry biomass, i.e., 70%, 54%, and 75%, respectively (at 100 mg/kg).	Application of Si NPs significantly improved the contents of chlorophyll and ameliorated oxidative stress by lowering the content of MDA, H_2_O_2_, and electrolyte leakage.	[65]
Fe (25, 50, and 100 mg/kg)	*T. aestivum* L.	Application of Fe NPs increased plant height, spike length, and dry weight under drought stress over control.	Chlorophyll a content increased up to 66% in wheat plants compared to the respective controls and eradicated the oxidative stress by switching antioxidative defense system.	[30]
ZnO (1.0%)	*T. aestivum* L.	Application of ZnO NPs strongly alleviated the delay in panicle initiation time and significantly increased grain yield as compared to the control under drought stress.	ZnO NPs enhanced Zn uptake but could not mitigate the negative impacts of drought stress on N and P uptake in wheat plants.	[104]
ZnO (50, 100, and 150 mg/L) and Si (150 and 300 mg/L)	*Mangifera indica* L.	Increased leaf area improved the total yield and fruit physiochemical characteristics as compared to the control under both NPs treatments.	Application of both NPs enhanced leaf NPK content; total carbohydrates, total sugars, and proline content; and SOD, POX, and CAT activities (at 100 mg/L nZnO and 150 mg/L nSi) over the control.	[109]
ZnO (0, 25, 50, and 100 mg/L)	*T. aestivum* L.	Application of ZnO NPs improved *T. aestivum* L. growth and crop yield.	Foliar spray enhanced chlorophyll content, and the activities of SOD and POXs.	[110]
SiO_2_ NPs, Se NPs, and SiO_2_/Se NPs (50 and 100 mg/L)	*Fragaria* × *ananassa* Duch.	Spraying of different NPs improved the growth and yield parameters of drought-stressed strawberry plants.	Treatment of Se/SiO_2_ at 100 mg/L showed maximal benefits to plants by preserving more photosynthetic pigments comparatively and increased relative water content, membrane stability index, and water use efficiency. Spraying of Se/SiO_2_ also increased drought tolerance via increasing the activities of CAT, APX, GPX, and SOD and decreased lipid peroxidation and H_2_O_2_ content.	[105]
Zero-valent copper NPs (3.333, 4.444, and 5.556 mg/L)	*Zea mays*	Cu NPs improved the biomass of drought-stressed plants, and increased total seed number and grain yield of maize plants.	Treatment of Cu NPs increased the contents of anthocyanin, chlorophyll, and carotenoid and improved drought stress tolerance by decreasing the oxidative stress via the enhancement of ROS scavenging antioxidative enzymes.	[106]
ZnO NPs (50 and 100 ppm)	*Solanum melongena* L.	Foliar spray of ZnO NPs improved the plant growth and productivity.	Application of ZnO NPs improved macro- and micronutrients’ uptake and increased relative water content in plants under drought stress.	[107]

**Table 4:** Some recent studies on the use of nanoparticles influencing the antioxidant defense system of the plant.

Nanoparticles	Plant Name	Biochemical Changes/Significant Findings	References
Ag	*Pennisetum glaucum*	Seed priming with Ag NPs increased the activity of antioxidant enzymes, proline content, total phenolics and flavonoid contents in pearl millet. Ion homeostasis is maintained by decreasing the sodium (Na^+^) and Na^+^/K^+^ ratio, while potassium (K^+^) increased by NPs leading to increased salt tolerance.	[147]
Si	*Coriandrum sativum*	Si NPs are found to have a beneficial effect on coriander under Pb stress. Foliar application of Si NPs alleviated the adverse impacts of Pb by modulating the vitamin C, flavonoids, antioxidant enzyme activities (CAT, POD, and SOD), and malondialdehyde (MDA) and by minimizing the oxidative stress.	[18]
Co_3_O_4_	*Brassica napus*	Low concentrations of Co_3_O_4_ NP treatment induced a beneficial effect on growth parameters by modulating APX, SOD, CAT, GR, phenylalanine ammonia lyase (PAL), tyrosine ammonia lyase (TAL), polyphenol oxidase (PPO), Guaiacol peroxidase (GPX), and glutathione S-transferase (GST) activity.	[148]
Fe_3_O_4_	*Zea mays*	A beneficial role of NPs on root development and membrane integrity is reported. Root length of maize plant significantly increased after NP treatment, with decreased malondialdehyde (MDA) level. NPs was also found to inhibit the pathways related to antioxidant defense.	[149]
ZnO	*Solanum lycopersicum*	Application of ZnO NPs led to increased tolerance in tomato plant towards *Tomato mosaic virus* (ToMV). Treatment of NPs led to increased growth parameters; photosynthesis; increases in the activity of enzymatic antioxidant defense systems (CAT, SOD, POX, APX, GR, and lipoxygenase (LOX) activity); and the accumulation of phenolic compounds, ascorbic acid content, and proline production.	[150]
Al_2_O_3_ and NiO	*Nigella arvensis*	Activity of antioxidant enzymes such as APX, CAT, SOD, and POD are found to increase. Total antioxidant capacity, reducing power, iridoids content, saponin content, and phenolic content are also found to be higher in plants treated with Al_2_O_3_ NPs (100–2500 mg/L) and low concentrations (<100 mg/L) of NiO NP treatment. However, at higher treatment level (>100 mg/L) of NiO NPs, concentrations of these metabolites were reduced.	[151]
CuO and S-nitrosoglutathione (GSNO)	*Lactuca sativa*	Treatment resulted in positive effects on plant growth and threefold increases in lettuce dry weight. Total phenolic content (two-fold) and flavonoid content (four-fold) were also increased significantly. Increased accumulation of micronutrients such as K, Na, Ca, Mg, and S is observed in the leaves.	[152]
Zn	*B. napus*	Many parameters such as protein, proline, total soluble sugar (TSS), total flavonoid content (TFC), and total phenolic content (TPC) were changed after NP treatment. Activity of antioxidant enzymes such as SOD, POD, and CAT also increased.	[153]
ZnO	*Vigna mungo*	Treatment was found to induce the activities of antioxidant enzymes such as GPX, GR, and CAT and was increase in the ascorbic acid and hydrogen peroxide contents.	[154]
Combination of FeO and hydrogel	*Oryza sativa*	Treatment alleviated the Cd and drought stress in rice plants. Rice plants were found to have increased biomass, increased activity of antioxidant enzymes (SOD, POD, and CAT), and increased photosynthetic efficiency, together with a reduction in ROS.	[60]
